# Long‐Term Effectiveness of Once‐Weekly Semaglutide in Patients With Type 2 Diabetes Previously Treated With Insulin. A Multicentre Real‐World Study

**DOI:** 10.1002/dmrr.70045

**Published:** 2025-04-25

**Authors:** Benedetta Maria Bonora, Andrea Giaccari, Agostino Consoli, Fabio Broglio, Angelo Avogaro, Gian Paolo Fadini, for the GLIMPLES Study Investigators

**Affiliations:** ^1^ Department of Medicine University of Padova Padua Italy; ^2^ Veneto Institute of Molecular Medicine Padua Italy; ^3^ Endocrine and Metabolic Center Fondazione Policlinico Universitario A. Gemelli IRCCS and Università Cattolica del Sacro Cuore Rome Italy; ^4^ Endocrinology and Metabolism Unit ASL Pescara Italy; ^5^ Department of Medicine and Aging Sciences DMSI and Center for Advanced Studies and Technology CAST “G. D'Annunzio” University of Chieti‐Pescara Chieti Italy; ^6^ Division of Endocrinology Department of Medical Sciences University of Turin Turin Italy

**Keywords:** body weight, glucose control, real‐world effectiveness, retrospective study, semaglutide, type 2 diabetes

## Abstract

**Background:**

The introduction of glucagon‐like peptide 1 receptor agonists (GLP‐1RAs) has provided new avenues for managing type 2 diabetes (T2D), aiming to achieve optimal glycaemic control while minimising treatment burden. We conducted a multicentre retrospective real‐world study to assess the effectiveness of semaglutide once‐weekly (OW) in patients previously treated with insulin.

**Methods:**

We included individuals with T2D who were on insulin (basal and/or bolus) and initiated OW semaglutide at 18 specialist care centres. We collected retrospective data on baseline clinical characteristics and updated values of HbA1c and body weight. The primary outcome was the change in HbA1c analysed using the mixed model for repeated measures. Secondary outcomes included the changes in body weight, insulin discontinuation and the change in insulin doses.

**Results:**

The study included 674 individuals. At baseline, participants were 61.7 years old, with a mean diabetes duration of 11.5 years and an HbA1c of 8.2%. During a median follow‐up of 18 months, OW semaglutide initiation led to a significant reduction in HbA1c (−0.9%) and body weight (−4.3 kg), with 60% of patients achieving HbA1c < 7%. 32.8% of patients discontinued insulin therapy, 72.5% of whom achieved an HbA1c < 7%. Among patients on basal‐bolus insulin, 75% completely discontinued bolus, 62% of whom achieved an HbA1c < 7%. Predictors of insulin discontinuation included shorter diabetes duration, lower baseline HbA1c, and lower insulin doses. Among patients who remained on insulin, initiation of OW semaglutide was associated with a decrease in total daily insulin requirement.

**Conclusion:**

Our study highlights OW semaglutide as a valuable addition to a T2D regimen based on insulin, offering effective glycaemic and weight control with the potential for insulin deintensification or discontinuation.

## 1 Introduction

Achieving good glycaemic control remains the main goal in treating type 2 diabetes (T2D) to prevent or reduce the risk of long‐term complications [1]. The range of glucose lowering medications (GLM) available to control hyperglycaemia has expanded significantly [2]. However, due to the progressive nature of the disease, many individuals eventually receive intensification with basal insulin or multiple daily injections (MDI) of insulin [3, 4]. These strategies increase the risk of hypoglycaemia, weight gain and the burden of blood glucose self‐monitoring and therapy self‐management, which negatively impact quality of life and adherence [5]. Despite insulin therapy, many individuals struggle to reach and maintain their HbA1c targets [6, 7]. Electronic chart data from 295 diabetes specialist care centres in Italy showed that despite treatment with insulin, 16.1% of patients have an HbA1c ≥ 9.0% [8]. Modern algorithms for the management of T2D prioritise glucagon‐like peptide 1 receptor agonist (GLP‐1RA) as the first injectable therapy whenever possible [9]. GLP‐1RAs lower plasma glucose concentrations with low risk of hypoglycaemia, promote weight loss, and improve blood pressure and lipid profiles [10]. Unlike insulin, GLP‐1RAs have a demonstrated ability of reducing major cardiovascular events, cardiovascular mortality and the risk of all‐cause mortality, both in primary and secondary prevention, along with renal protective effects [11]. A meta‐analysis of randomized controlled trials (RCT) comparing GLP‐1RA versus insulin has shown that long‐acting GLP‐1RA has greater effects on HbA1c reduction than basal insulin [12]. Furthermore, GLP‐1RA treatment led to a significant weight loss, whereas insulin therapy was associated with weight gain. Compared with insulin, GLP‐1RA treatment also significantly reduces cardiovascular risk factors such as blood pressure, LDL cholesterol and triglycerides. Proportions of patients reporting hypoglycaemic episodes were significantly lower with GLP‐1RA compared with insulin treatment [12]. Real‐world studies (RWS) have consistently confirmed the broad effectiveness of GLP‐1RA in reducing HbA1c and body weight across patient subgroups [13].

Compared to rapid‐acting insulin in patients with a background regimen including oral agents and basal insulin, GLP‐1RA allowed a similar or greater effect on HbA1c, greater weight loss and a lower incidence of hypoglycaemic events [12, 14, 15].

Previous randomized controlled trials have demonstrated that a fixed or loose combination of GLP‐1RA and basal insulin significantly reduces HbA1c and body weight in patients with uncontrolled T2D [16]. RWS suggest that initiating GLP‐1RA often allows for deintensification of the MDI insulin regimen [17, 18]. The latest ADA standard of care recommends simplifying or de‐intensifying complex insulin regimens in some people with clinical complexity and/or treatment burden to decrease the risk of hypoglycaemia, increase patient acceptance, and improve quality of life [2].

Among the available GLP‐1RA, once‐weekly (OW) semaglutide has been identified as the most potent in reducing HbA1c and body weight [19]. In a dedicated cardiovascular outcomes trial, OW semaglutide has shown the capacity to reduce major adverse cardiovascular events and with reduced rates of renal endpoints [20, 21]. Therefore, initiating OW semaglutide in patients with T2D who are already receiving insulin could improve glycaemic control and allow therapeutic simplification.

Herein, we present the results of a multi‐centre retrospective study on the effectiveness of initiating OW semaglutide in patients previously treated with insulin, providing fresh insights into the opportunity of re‐positioning patients with T2D according to the modern therapeutic algorithm.

## 2 Methods

### 2.1 Study Design

GLIMPLES (GLp‐1 for therapeutic sIMPLification in type 2 diabetES) was a multicentre retrospective RWS collecting electronic chart data from 18 diabetes specialist care centres in Italy.

The protocol was first approved by the Ethical Committee of the coordinating centre (University Hospital of Chieti‐Pescara, prot. n. 09 dated 19/05/2022) and by the Ethical Committees of all participating centres. The study was conducted in accordance with the Declaration of Helsinki. Data were extracted automatically from the same electronic chart system (MetaClinic, Me.Te.Da, San Benedetto del Tronto, Italy). There was no possibility of re‐identifying individuals based on the retrieved data. Since data were anonymised at the time of automatic extraction, no informed consent was required according to national regulations. The data collection procedure has been described before [22, 23].

### 2.2 Definition of Exposure

All patients who initiated GLP‐1RA for the first time between 1 January 2010 and 31 December 2021 were included in the database. The available GLP‐1RA were exenatide bis in die (BID), exenatide OW, liraglutide, lixisenatide, dulaglutide, OW semaglutide and oral semaglutide. The index date was defined as the day on which patients received a prescription for the index drug.

No information on drug dispensation or refill rates was available, so it was impossible to evaluate medical adherence. Persistence on treatment was defined based on continuous prescription of the index drug at subsequent visits.

In the present study, we analysed only data on patients previously treated with insulin who initiated OW semaglutide, with the aim of describing effectiveness on HbA1c and body weight, as well as changes in insulin doses and the probability for insulin discontinuation.

Inclusion criteria were: age 18 or older, a diagnosis of T2D done at least 1 year before index date as recorded in the chart, initiation of OW semaglutide, treatment with any insulin in the prior visit and no treatment with any other GLP‐1RA in the prior visits. Exclusion criteria were: age < 18; diabetes other than type 2, and lack of follow‐up data in the electronic chart at least for one of the endpoints described below.

### 2.3 Variables

For all patients, we collected the following information: age, gender, body weight, height and body mass index (BMI), systolic and diastolic blood pressure, diabetes duration, fasting glucose, HbA1c, smoking habit, lipid profile (total, HDL and LDL cholesterol, and triglycerides), serum creatinine, eGFR (calculated using the CKD‐EPI equation), urinary albumin to creatinine ratio (UACR), ongoing medications for the management of diabetes and cardiovascular risk factors. We also obtained information on chronic complications of diabetes as follows: diabetic retinopathy defined based on digital fundus photography scored by expert ophthalmologists; stage III or higher chronic kidney disease (CKD), defined as an eGFR of 60 mL/min/1.73 m^2^ or lower; UACR ≥ 30 mg/g; somatic peripheral and autonomic neuropathy; history of foot problems; coronary heart disease (CHD) defined as either myocardial infarction, angina, instrumental evidence of cardiac ischaemia or coronary revascularisation; carotid atherosclerosis or history of transient ischaemic attack or stroke; peripheral arterial disease defined as significant stenosis in leg arteries or history of revascularisation.

The dose of OW semaglutide at each visit after the index date was recorded along with the information on whether the prescription was confirmed or not.

### 2.4 Endpoints

The primary study endpoint was the change in HbA1c from baseline through follow‐up visits with confirmed prescription for OW semaglutide. The main secondary endpoint was the change over time in body weight.

We also compared the changes in HbA1c and body weight between patients who discontinued semaglutide during follow‐up and those who continued semaglutide. Additionally, we examined these endpoints among patients who had stopped their insulin regimen compared with those who continued insulin. Finally, we evaluated insulin deintensification and withdrawal, and the achievement of HbA1c below 7%.

### 2.5 Statistical Analysis

Continuous variables are expressed as mean and standard deviation, whereas categorical variables are presented as numbers and percentages. Comparison between the two groups was performed using Student's *t* test for continuous variables and the chi‐squared test for categorical variables. The change over time in HbA1c and body weight were estimated using the mixed model for repeated measures. Time, baseline HbA1c and persistence on drug were entered as fixed factors. The first order autoregressive covariance was used, and the model output was estimated means at each time point along with 95% confidence interval. The same analysis was repeated for body weight. A univariate logistic regression analysis was performed to identify variables associated with a positive response to OW semaglutide in achieving an HbA1c < 7%. A multivariable Cox proportional hazard regression model was used to evaluate the predictors of insulin withdrawal. The risk function for insulin discontinuation was calculated using coefficients and models derived from our previous study [17]. Statistical significance was set at the conventional 5% type 1 error. SPSS version 28 was used for all analyses.

## 3 Results

### 3.1 Patient Population

**FIGURE 1:**
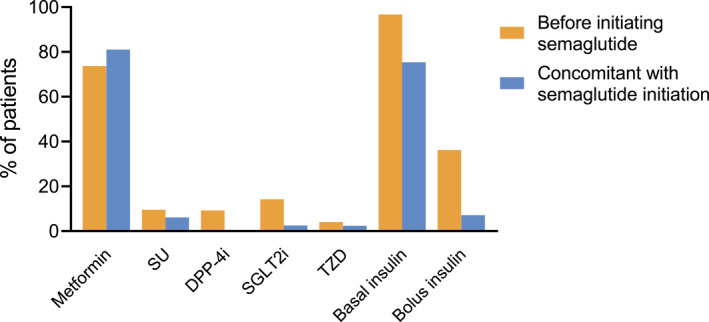
Changes in diabetes treatment regimen at the time of OW semaglutide initiation. All differences were statistically significant (*p* < 0.05).

Baseline characteristics of the 674 individuals who initiated OW semaglutide and had at least one available follow‐up examination after baseline are summarised in Table 1. Participants were, on average, 61.7 years old, with a known diabetes duration of 11.5 years, and 64% were male. The baseline BMI was 32.4 kg/m^2^ and HbA1c was 8.2% (66 mmol/mol). At the time of OW semaglutide initiation, 20.3% of patients had an HbA1c below 7%. Regarding diabetes complications, 44.2% of patients had at least one microangiopathy and 50.3% had macroangiopathy. Baseline eGFR was 80.3 mL/min/1.73 m^2^, 21.3% had CKD stage III or higher, and 37.6% had micro‐ or macroalbuminuria. Before the index date, all patients were on insulin according to inclusion criteria. The regimen after adding OW semaglutide included metformin (81%), basal insulin (75.4%), sulphonylurea (6.1%), pioglitazone and SGLT‐2 inhibitors (2.4%). A small percentage (7.1%) of patients were concomitantly treated with MDI insulin. 23.6% of patients withdrew insulin at the index date, that is, at the time of semaglutide initiation. The change in diabetes treatment regimen at the time of OW semaglutide initiation is shown in Figure 1.

**TABLE 1:** Characteristics of study patients.

	Value
Demographics	
Male sex, %	63.8
Age, years	61.7 (9.8)
Diabetes duration, years	11.5 (8.9)
Clinical and laboratory data	
Body weight, kg	93.2 (17.6)
Body mass index, kg/m^2^	32.4 (5.7)
Systolic blood pressure, mm Hg	141.3 (20.1)
Diastolic blood pressure, mm Hg	81.2 (10.6)
Fasting glucose, mg/dL	157.9 (60.8)
HbA1c, %	8.2 (1.6)
HbA1c, mmol/mol	65.9 (17.5)
Total cholesterol, mg/dL	165.8 (42.0)
HDL cholesterol, mg/dL	44.2 (11.9)
LDL cholesterol, mg/dL	91.2 (36.3)
Triglycerides, mg/dL	159.0 (94.0)
eGFR, mL/min/1.73 m^2^	80.3 (23.3)
UACR, mg/g	160.9 (795.7)
Complications	
CKD stage III+, %	21.3
Albuminuria > 30 mg/g, %	37.6
Retinopathy, %	27.9
Coronary heart disease, %	23.8
Established CVD, %	25.1
Any microangiopathy, %	44.2
Any macroangiopathy, %	50.3
Diabetes medications	
Metformin, %	73.7
Sulphonylureas, %	9.5
DPP‐4 inhibitors, %	9.2
SGLT‐2 inhibitors, %	14.2
Pioglitazone, %	4.0
Basal insulin, %	96.7
Bolus insulin, %	36.2
Other therapies	
Statin, %	66.8
Anti‐platelet agents, %	45.5
RAS blockers, %	60.8
Beta‐blockers, %	34.4
Calcium channel blockers, %	26.4
Diuretics, %	32.4
Anticoagulants, %	2.4

*Note:* Data are presented as mean (standard deviation) or as percentage. Percentage availability is indicated for each variable.

Abbreviations: CKD, chronic kidney disease (defined as eGFR < 60 mL/min/1.73 m^2^); eGFR, estimated glomerular filtration rate; RAS, renin angiotensin system; SGLT‐2, sodium glucose co‐transporter‐2; UACR, urinary albumin excretion rate.

The last recorded dose of OW semaglutide was 0.25 mg in 9%, 0.50 mg in 46.9% and 1 mg in 44.1% of patients.

### 3.2 Change in HbA1c

The median follow‐up time was 18 months (IQR 12–27) with a maximum span of 45 months. During the observation period, the prescription of semaglutide was discontinued in 28.3% of patients. The most common regimens initiated after discontinuation of semaglutide were: other GLP‐1RAs (12% dulaglutide, 12% oral semaglutide, 11% liraglutide), SGLT‐2 inhibitors (37.2%) and bolus insulin (32.5%).

**FIGURE 2:**
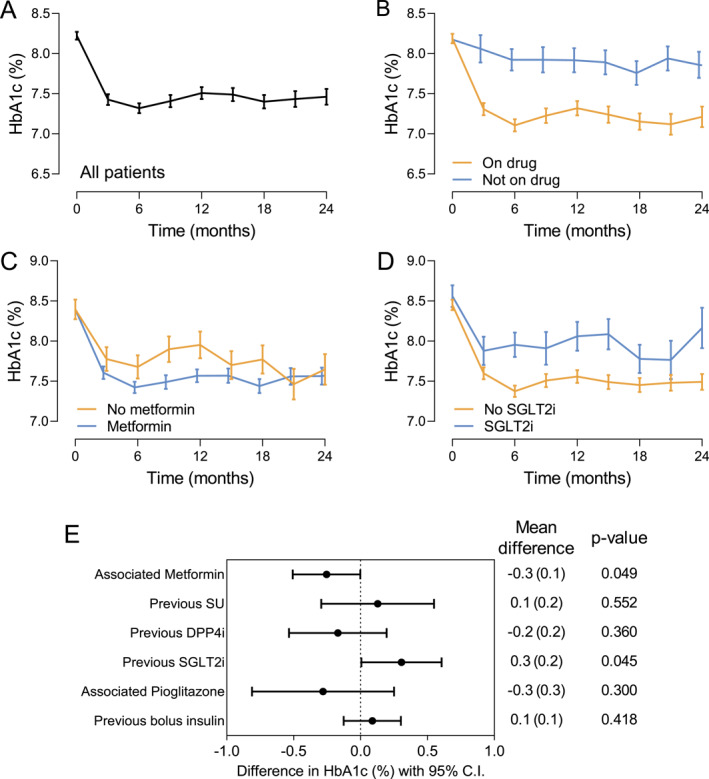
Effectiveness on HbA1c. (A) Change in HbA1c from baseline to 24 months in all patients. (B) Change in HbA1c from baseline to 24 months in patients who continued treatment with OW semaglutide and in patients who discontinued the treatment. (C) Change in HbA1c from baseline to 24 months in patients concomitantly treated with metformin or not. (D) Change in HbA1c from baseline to 24 months in patients with a prior treatment with SGLT‐2 inhibitors or not. Data are shown as mean and bars indicate standard error. (E) Forest plot showing the effect of concomitant or previous treatments (yes vs. no) as mean difference and 95% confidence interval (CI).

The change over time in HbA1c and body weight was estimated using the mixed model for repeated measures, which considers all patients contributing with at least one value at baseline or during observation. The maximum estimated mean (SD) change in HbA1c, adjusted for baseline HbA1c, was −0.9% (0.1%) at 6 months and the mean difference during observation was −0.8% (*p* < 0.001; Figure 2A). The estimated change in HbA1c was significantly higher in patients who continued treatment with OW semaglutide during the observation compared with those who discontinued treatment (mean difference between groups −0.6%, *p* < 0.001; Figure 2B). There were no significant differences between men and women in HbA1c change among patients treated with OW semaglutide (data not shown). The reduction in HbA1c was significantly greater in patients who were concomitantly treated with metformin than in those who were not (mean difference −0.3%, *p* = 0.049; Figure 2C). On the other hand, a prior treatment with SGLT2 inhibitors was associated with less prominent HbA1c improvement by 0.3% (*p* = 0.045; Figure 2D), likely because the SGLT‐2 inhibitors were discontinued in most cases at the time of OW semaglutide initiation (Figure 1). Other previous or concomitant treatments did not modify the glycaemic response after initiating semaglutide (Figure 2E).

During the observation, 60% of patients who had a baseline HbA1c above 7.0% and continued the treatment achieved an HbA1c value below 7.0%, and 24% achieved an HbA1c value below 6.5%. Among patients who discontinued semaglutide, only 24% achieved a reduction in HbA1c below 7%.

Variables significantly associated with achieving an HbA1c below 7% were: shorter diabetes duration, lower body weight at baseline, and absence of microangiopathy (Table 2).

**TABLE 2:** Variables associated with achieving an HbA1c < 7%.

Variables	OR (95% CI)	*p*‐value
Age, years	0.985 (0.968–1.003)	0.100
Sex, male	0.809 (0.568–1.153)	0.241
Diabetes duration, years	0.969 (0.950–0.988)	0.001
Baseline HbA1c, %	1.036 (0.927–1.157)	0.532
Baseline body weight, kg	0.988 (0.978–0.998)	0.021
Presence of microangiopathy	0.593 (0.418–0.839)	0.003
Presence of macroangiopathy	0.813 (0.565–1.168)	0.262
Use of metformin	1.342 (0.862–2.089)	0.192
Total insulin dose, IU	0.999 (0.991–1.007)	0.772

*Note:* A univariate logistic regression analysis was performed to identify variables associated with a positive response to OW semaglutide in achieving an HbA1c < 7%.

### 3.3 Body Weight Change and Other Endpoints

**FIGURE 3:**
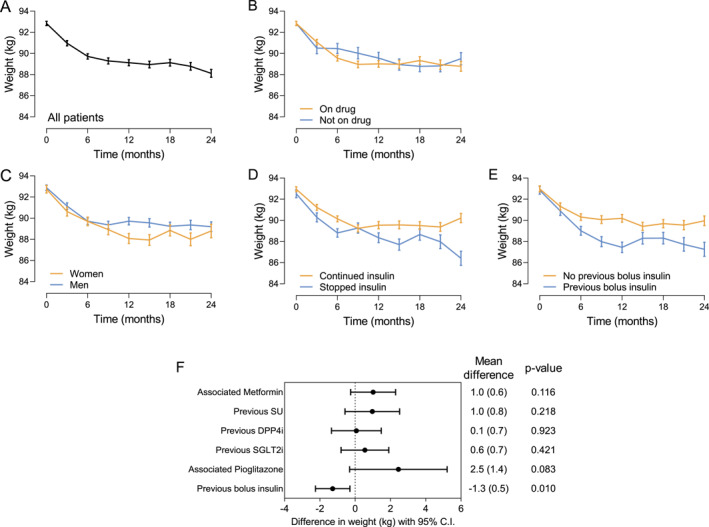
Effectiveness on body weight. (A) Change in body weight from baseline to 24 months in all patients. (B) Change in body weight from baseline to 24 months in patients who continued treatment with OW semaglutide and in patients who discontinued the treatment, often switching to other glucose‐lowering medications. (C) Change in body weight from baseline to 24 months in men versus women. (D) Change in body weight from baseline to 24 months in patients who stopped insulin treatment and in patients who continued insulin treatment. (E) Change in body weight from baseline to 24 months in who were previously treated with bolus insulin and in those who were not. Data are shown as mean and bars indicate standard error. (F) Forest plot showing the effect of concomitant or previous treatments (yes vs. no) as mean difference and 95% confidence interval (CI).

The maximum estimated mean (SD) change in body weight adjusted for baseline weight was −4.3 kg (0.5 kg) at 21 months, with a mean difference during observation of −3.5 kg, (*p* < 0.001; Figure 3A), and 58.0% of patients experienced a body weight reduction of 5% or greater their initial body weight. 19% of subjects achieved weight loss ≥ 10%. The reduction of body weight appeared not significantly different in patients who continued treatment with OW semaglutide compared with those who discontinued treatment (mean difference −0.5 kg; *p* = 0.259; Figure 3B). Women treated with OW semaglutide experienced a significantly greater weight loss during follow‐up compared with men (mean difference −1.8 kg, *p* < 0.001; Figure 3C). The estimated reduction in body weight was significantly lower in patients who remained on insulin regimen (mean difference −2.1 kg, *p* < 0.001; Figure 3D) and was significantly greater by 1.3 kg in patients who were previously treated with bolus insulin (*p* = 0.006; Figure 3E), likely because bolus insulin was mostly discontinued at the time of semaglutide initiation. The change in body weight was not associated with the class of drugs initiated after discontinuation of OW semaglutide (Figure 3F).

At 24 months after index date, systolic blood pressure significantly declined by 4.3 mm Hg (95% CI −7.9 to −0.6 to mm Hg) and total cholesterol significantly declined by 14.7 mg/dL (95% CI −20.7 to −8.8 mg/dL).

### 3.4 Insulin Deintensification and Predictors of Insulin Discontinuation

At the end of the observation period, 221 subjects (32.8%) had completely stopped insulin, either basal or bolus. Among these patients, 72.5% who had a baseline HbA1c value above 7.0% achieved an HbA1c value below 7.0%. Among patients who remained on insulin, only 149 individuals achieved an HbA1c value below 7.0%, equal to 40.4% of those who had a baseline HbA1c value above 7.0%.

**FIGURE 4:**
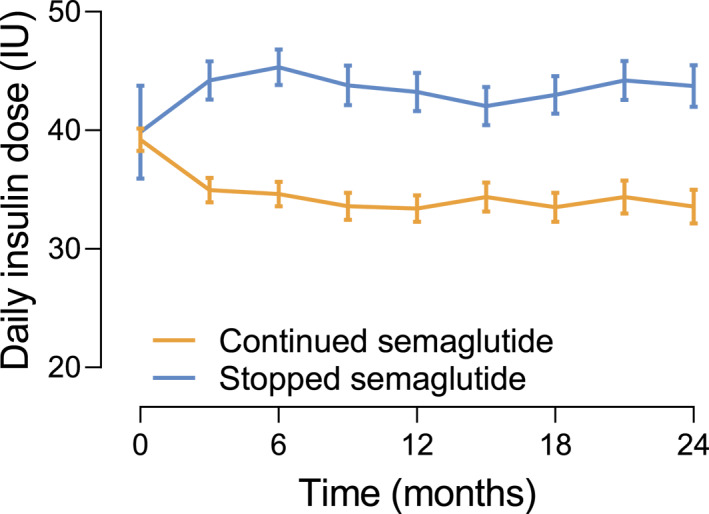
Effectiveness of treatment with OW semaglutide on insulin dose in patients who continued insulin regimen. The change in insulin dose HbA1c from baseline to 24 months in patients who continued treatment with OW semaglutide and in patients who discontinued the treatment. Data are shown as mean and bars indicate standard error.

In patients who remained on insulin, the treatment with OW semaglutide was associated with a significant reduction in insulin doses that persisted over the time (mean difference 10.0 UI, *p* < 0.001; Figure 4).

Of the 248 patients treated with MDI before the index date, 186 (75%) completely discontinued bolus insulin during the follow‐up period. Of these patients, 62% who had a baseline HbA1c value above 7.0% achieved an HbA1c value below 7.0%. In contrast, only 27.4% of subjects who remained on the MDI regimen achieved the target of HbA1c below 7.0%.

Variables significantly associated with insulin discontinuation in the multivariate Cox proportional hazard model were shorter diabetes duration, lower HbA1c at baseline, absence of microangiopathy and lower baseline insulin doses (Table 3). We calculated the risk function for insulin withdrawal using the model coefficients obtained from our previous study [17]. The model included the following baseline variables: age, diabetes duration, HbA1c, presence of microangiopathy, use of metformin, total insulin dose and duration of observation. The discrimination analysis yielded C‐statistics of 0.74 (95% CI 0.70–0.78; *p* < 0.001) for insulin discontinuation.

**TABLE 3:** Predictors of insulin discontinuation.

Covariate	*B* (SE)	*p*‐value
Age, years	0.004 (0.008)	0.572
Diabetes duration, years	−0.088 (0.012)	< 0.001
Baseline HbA1c, %	−0.117 (0.036)	0.001
Presence of microangiopathy	−0.492 (0.163)	0.002
Use of metformin	0.348 (0.230)	0.141
Total insulin dose, IU	−0.027 (0.006)	< 0.001
Sex (male)	0.086 (0.143)	0.547

*Note:* A multivariable Cox proportional hazard model was used to identify the independent predictors of the insulin discontinuation.

## 4 Discussion

This RWS from the Italian specialist diabetes care shows that adding OW semaglutide in patients already treated with insulin regimen is associated with significant HbA1c reduction, bringing 60% of patients who had HbA1c > 7% down to the conventional HbA1c target of 7%. This reduction is accompanied by weight loss and improvements in cardiovascular risk factors along with simplification of treatment regimen.

Our findings support the growing body of evidence on the use of GLP‐1RAs as an ideal therapeutic option in T2D management, even in patients already receiving insulin [9, 10]. GLP‐1RA offers several advantages over insulin therapy, including a lower risk of hypoglycaemia, significant weight loss and favourable effects on cardiovascular risk factors (including blood pressure and lipids) and cardiovascular and renal outcomes [10, 11].

It is worth noting that these results were achieved with less than the maximal dose of OW semaglutide in half of the patients. Indeed, at the end of the observation, roughly 1 out of 2 patients were receiving the 0.5 mg maintenance dose and only 44% had escalated to the 1 mg dose, the maximum dose available in Italy. However, a small proportion of patients remained on the starting 0.25 mg dose, which is not considered a maintenance dose of OW semaglutide. We do not have information on whether the incomplete dose escalation was due to gastrointestinal side effects or clinical inertia. Other possible explanations include the physician deciding to maintain the 0.5 mg dose after the patient had reached their HbA1c target or that the follow‐up time for some patients ended before dose escalation was completed. Targeting the 1 mg dose in all patients, as done in RCTs, would have allowed even greater improvements in glycaemic control, body weight, and insulin discontinuation [24].

Observed reductions in HbA1c among patients initiating OW semaglutide were slightly lower than those observed in SUSTAIN RCTs [24] but consistent with other RWS [25, 26, 27, 28, 29, 30, 31]. It is important to note that unlike RCTs, patients in our study were older, had a longer duration of diabetes and 20% had a baseline HbA1c below 7%. Moreover, since our population consisted of insulin‐treated patients with a long duration of diabetes, we cannot rule out the possibility that a small percentage of undiagnosed LADA cases may be present among them. This may have contributed to the heterogeneity of the results regarding HbA1c reduction.

The reduction of 4 kg in body weight was consistent with previous RCTs and RWS [24, 25, 26, 27, 28, 29, 30, 31]. The lack of difference in body weight between those who continued and those who discontinued OW semaglutide may be attributable to the fact that most patients switched to another GLP‐1RA or SGLT‐2 inhibitor.

Consistent with results reported in cohort studies and post hoc analysis of RCT with exenatide BID, OW exenatide and dulaglutide [32], we found that women treated with OW semaglutide exhibited, on average, greater weight loss compared with men. The mechanism behind this difference remains unclear. It has been hypothesised that the higher drug exposure observed in women, possibly due to their lower average body weight, may play a role [33].

Previous RCTs have demonstrated that GLP‐1RA had a similar or greater effect on HbA1c and greater weight loss with a lower incidence of hypoglycaemic events compared to rapid‐acting insulin in patients with a background basal insulin regimen plus GLM [12, 14, 15]. Two RCTs have previously provided evidence that it is possible and safe to switch from a MDI insulin regimen to either a single daily fixed combination or a loose combination of GLP‐1RA and basal insulin [34, 35]. In both trials, patients randomized to receive GLP‐1RA experienced a similar reduction in HbA1c compared with patients assigned to the intensification of MDI insulin regimen. The proportion of patients achieving HbA1c ≤ 7% was not significantly different between the two groups. Patients who received GLP‐1RA showed a significant reduction in body weight, a decrease in mean total insulin dose, and fewer episodes of hypoglycaemia [34, 35]. However, in the trial with albiglutide, only 54% of participants were able to completely replace rapid insulin with albiglutide through week 26 [35].

Our results underscore the importance of exploring therapeutic options beyond traditional insulin regimens to achieve optimal glycaemic control while minimising treatment burden and associated adverse effects. Importantly, our study provides insights into the potential for insulin deintensification with the introduction of OW semaglutide. Three out of 4 patients had stopped prandial insulin, and a substantial proportion of patients were able to withdraw insulin therapy and achieve optimal glycaemic targets. Patients who withdrew insulin showed improvements in glycaemic control, allowing 72.5% of those with a baseline HbA1c above 7.0% to achieve an HbA1c value below 7.0%. Overall, the potential for insulin deintensification or withdrawal with the introduction of a GLP‐1RAs aligns with prior observational studies [17, 18]. We previously demonstrated the possibility of discontinuing prandial insulin in 58.6% of patients who were on MDI insulin regimen after the initiation of a GLP‐1RA. Patients who discontinued prandial insulin experienced improvements in HbA1c and body weight similar to what we observed in this study [17]. Notably, the withdrawal of insulin therapy was associated with specific clinical features, including shorter diabetes duration, lower baseline HbA1c levels and lower insulin requirement, highlighting the importance of an individualised treatment strategy tailored to patient characteristics. The baseline clinical characteristics that predicted insulin withdrawal were consistent with those identified in our previous observational study [17].

Instead, in patients who remained on insulin regimen, the addition of OW semaglutide was associated with a decrease in mean total insulin dose, consistent with findings from previous studies [15, 26, 35].

### 4.1 Strengths and Limitations

Our study has several strengths. First, subjects were followed for a mean of 18 months after initiation of OW semaglutide. To date, the effects of OW semaglutide on glycaemic control in routine clinical practice have been reported for up to 12 months [25, 28, 31] with only two RWS having a prolonged observation period up to 2 years [26, 27]. In the study by Vilsbøll et al., the discontinuation rate was quite high, up to 43.9% at 2 years [26]. Second, our results are based on data retrieved from 18 diabetes specialist care centres in Italy, representing a diverse population from routine clinical practice.

The study has intrinsic limitations due to its observational design. Data were collected as part of routine clinical practice, which may have affected the robustness and completeness of the data. We do not have information about changes in patients' lifestyle habits during the follow‐up. Changes in diet or exercise could have influenced the results for HbA1c, body weight, and lipid levels. Additionally, we lacked information on tolerability, adherence, and reasons for discontinuation of OW semaglutide, thereby providing limited insight into strategies to improve persistence. Also, there was no control group, such that it was not possible to compare new users of OW semaglutide with those who intensified insulin therapy. Furthermore, we acknowledge the absence of data on the duration of MDI insulin therapy, which might influence the likelihood of discontinuation. Lastly, not all patients had the same observation time and schedule of follow‐up.

## 5 Conclusion

The findings of this long‐term RWS support the role of OW semaglutide as a valuable addition to the treatment arsenal for T2D, even in patients already receiving insulin. OW semaglutide offers effective glycaemic control, weight reduction, improvement in cardiovascular risk factors, and the potential for insulin deintensification or withdrawal.

## Author Contributions

Study design: B.M.B., A.G., A.C., F.B., A.A., G.P.F. Data collection: B.M.B., A.C., A.A., G.P.F. Data analysis and interpretation: B.M.B., A.G., A.C., F.B., A.A., G.P.F. Manuscript writing: B.M.B., G.P.F. Manuscript revisions: A.G., F.B., A.C. and A.A. All authors approved the final version of the manuscript.

## Ethics Statement

The study was first approved by the Ethical Committee of the coordinating centre (University Hospital of Chieti‐Pescara, prot. n. 09 dated 19/05/2022) and by the Ethical Committees of all participating Centres.

## Conflicts of Interest

B.M.B. received lecture or advisory board fees from AstraZeneca, Boehringer‐Ingelheim, Lilly, MSD, Sanofi, Servier and Novo‐Nordisk. A.G. has received lecture fees from AstraZeneca, Lilly and Novo Nordisk. A.C. received grants from AstraZeneca, Lilly, Novo‐Nordisk. He also received speaker fees, and provided advisory board services for Abbott, AstraZeneca, Boehringer Ingelheim Pharmaceuticals, Lilly, Merck Sharp & Dhome, Menarini, Novo‐Nordisk, Sanofi, Sigma‐Tau and Takeda. F.B. received lecture fees from Lilly and Novo‐Nordisk. A.A. received research grants and lecture or advisory board fees from MSD, AstraZeneca, Novartis, Boehringer Ingelheim, Sanofi, Mediolanum, Janssen, Novo Nordisk, Lilly, Servier and Takeda. G.P.F. received advisory board grants, honoraria or lecture fees from Abbott, AstraZeneca, Boehringer, Lilly, MSD, Novartis, Novo Nordisk, Sanofi, Servier and Takeda.

### Peer Review

The peer review history for this article is available at https://www.webofscience.com/api/gateway/wos/peer-review/10.1002/dmrr.70045.

## Data Availability

Restrictions apply to the availability of source data used in this study, mainly for privacy policy. The datasets generated during the current study are not publicly available but may be available from the corresponding author upon reasonable request.
